# Impact of salt intake reduction on CVD mortality in Costa Rica: A scenario modelling study

**DOI:** 10.1371/journal.pone.0245388

**Published:** 2021-01-12

**Authors:** Jaritza Vega-Solano, Adriana Blanco-Metzler, Karol Madriz-Morales, Eduardo-Augusto Fernandes-Nilson, Marie Eve Labonté

**Affiliations:** 1 Costa Rican Institute of Research and Teaching in Nutrition and Health (INCIENSA), Tres Ríos, Cartago, Costa Rica; 2 Center for Epidemiological Research on Health and Nutrition, University of São Paulo, São Paulo, São Paulo, Brazil; 3 Centre Nutrition, Santé et Société (NUTRISS), Institute of Nutrition and Functional Foods (INAF), Université Laval, Québec, Canada; The Ohio State University College of Medicine, UNITED STATES

## Abstract

Cardiovascular diseases (CVD) represent the leading cause of death in Costa Rica and high blood pressure was associated with a mortality rate of 29% in 2018. The average household sodium intake in the country is also two times higher than the World Health Organization recommendation. The objective of this study was to estimate the impact of reducing salt intake on CVD mortality in Costa Rica using a scenario simulation model. The Preventable Risk Integrated ModEl (PRIME) was used to estimate the number of deaths that would be averted or delayed in the Costa Rican population by following the national and the international guidelines to reduce salt consumption, according to two scenarios: A) 46% reduction and B) 15% reduction, both at an energy intake of 2171 kcal. The scenarios estimated that between 4% and 13%, respectively, of deaths due to CVD would be prevented or postponed. The highest percentages of deaths prevented or postponed by type of CVD would be related to Coronary heart disease (39% and 38%, respectively), Hypertensive disease (32% and 33%, respectively), and Stroke (22% in both). The results demonstrate that reducing salt consumption could prevent or postpone an important number of deaths in Costa Rica. More support for existing policies and programs urges.

## 1. Introduction

Scientific evidence shows a direct relationship between excessive sodium intake and an increased risk of high blood pressure (HBP) and other cardiovascular diseases (CVD) [1].

The World Health Organization (WHO) considers sodium reduction interventions as "best-buys" for the population, since they likely represent the most cost-effective public health measures, according to evidence to reduce the global burden of non-communicable diseases (NCDs) [2–5].

Between 2012 and 2013, daily sodium intake in Costa Rica was 3.8 g per person for 2000 kcal, which is equivalent to 9.3 g/day of salt, therefore corresponding to almost twice the WHO maximum recommendation (2000 mg of sodium or 5 g of salt/day for 2000 kcal) [6, 7]. The main sources of sodium were discretionary salt (60%) and processed foods, including seasonings with added sodium (27%) [6].

NCDs represent the leading cause of death in Costa Rica. In 2018, they were responsible for 83% of total deaths, and 29% of these deaths corresponded to CVDs [8].

Moreover, the prevalence of HBP in the Costa Rican population over 19 years old was 36.2% in 2014, with an additional 24% of the population in the pre-hypertensive state [9].

In 2012, the cost of care for HBP patients in the country was higher than ¢47,308 million per year (about USD$ 80,000), and represented 3.5% of national social security expenditure [9].

Given the commitment of the Costa Rican government with PAHO´s “Policy Statement Preventing Cardiovascular Disease in the Americas by Reducing Dietary Salt Intake Population-Wide” [10] and the high prevalence and health care costs of HBP, the “National Plan for the reduction of salt/sodium consumption in Costa Rica 2011–2021” was created in 2011 [11]. Two years later, the Program for the Reduction of Salt/Sodium Consumption was officialized and declared of public and national interest [12].

In 2014, the Costa Rican government additionally created the “National Strategy for the Comprehensive Approach to Chronic Non-Communicable Diseases and Obesity 2014–2021”. The following goals were included in this national strategy: 1) 17% relative reduction of premature mortality due to diseases such as CVD, cerebrovascular diseases and hypertension; 2) 15% relative reduction of the average salt/sodium intake of the population and 3) Relative reduction of HBP prevalence in the country [13].

Global research indicates that a 10-year period of salt consumption reduction would prevent the loss of 5.8 million years of good health (DALYs) at a low cost [2]. Other WHO statistics estimate that reducing salt consumption to the level they recommend could prevent 2.5 million deaths every year [14].

A recent investigation was carried out to estimate the economic benefit for the Costa Rican Social Security System (CCSS, for its acronym in Spanish) of successfully implementing the “National Plan to Reduce Public Consumption of Salt/Sodium in Costa Rica 2011–2021” [11]. The CCSS is the institution responsible for providing comprehensive health care for Costa Ricans, which would be directly affected by the effectiveness of the National Plan. A projection for 2022–2031, under the assumption of a 5% reduction in the prevalence of HBP through a successful implementation of the National Plan, showed that significant cost savings related to the care of patients with HBP could occur [15].

Analyses based on simulation models are a reasonable option to synthesize results of observational studies where experimental studies are difficult to conduct. Such analyses help public health actors to make informed decisions in the absence of reliable data. However, studies about the impact of population-wide dietary interventions and policies on health and cost-effectiveness are scarce in Costa Rica, as is the evidence on the epidemiological burden of dietary risk factors, such as sodium consumption, on CVD [16–18].

Therefore, the objective of the current study was to estimate the impact of reducing salt intake on CVD mortality in Costa Rica using a scenario simulation model set in 2018. We hypothesized that reducing salt intake in the Costa Rican population would reduce mortality from CVD.

## 2. Materials and methods

### 2.1 The simulation model

The Preventable Risk Integrated ModEl (PRIME) was used in the present study to estimate the number of deaths due to CVDs that would be averted or delayed in the Costa Rican population by following the country's goal recommendations and the WHO guidelines to reduce salt consumption. PRIME is a macro-simulation model designed to estimate the impact of changes in the distribution of behavioral risk factors (including dietary factors such as salt intake) on mortality from various NCDs related to the risk factors of interest [19]. Links between behavioral risk factors and NCD mortality are parameterized in PRIME based on results of published meta-analyses of epidemiological studies [19]. To specifically estimate the deaths attributable to sodium consumption, the PRIME model uses the results from a meta-analysis of randomized controlled trials that link Hypertension to CVD [20].

PRIME requires the entry of age- and sex-specific estimates of the number of individuals living in the population under study as well as the age- and sex-specific estimates of the annual number of deaths from each relevant NCD in that population. It also requires the reference distribution (mean and standard deviation) of the risk factors of interest for the baseline and counterfactual scenarios (herein, salt intakes), usually determined using national survey data [19].

Following the entry of the collected baseline data in PRIME, the user can enter any desired hypothetical (counterfactual) scenario. The hypothetical scenarios consist of variations in the distribution of one or more specific behavioral risk factors as compared with the baseline scenario. Then, PRIME estimates the change in the annual number of NCD deaths between the baseline and counterfactual scenarios [16–19]. The PRIME application is available from one of its creators upon request.

### 2.2 Baseline scenario: Current salt intake in the Costa Rican population

Baseline intake of salt in the Costa Rican population was estimated from the Household Budget Survey (HBS) from 2012–2013 [6]. This indirect estimation methodology allowed to establish the average salt intake (g / day) for 2000 kcal and for the actual caloric intake (2171 kcal / day) of the overall population. Results per strata were grouped according to the recommendations of the developers of this methodology [21], and the standard deviation was calculated to allow the elimination of extreme values (S1 Table).

Table 1 presents the results for salt intake:

**Table 1:** Estimates of salt intake and caloric intake in the Costa Rican population in 2012–2013.

Energy intake (kcal/day)	Corresponding salt intake (g/day)
2000	9.0±5.0
2171	10.0±5.4

Data from 2012–2013 were used because these are the most up-to-date data on sodium intake available in the country. The latest National Nutrition Survey (ENN) in the country was carried out in 2009 [11]. The ENN 2009 and an analysis of sodium availability in Costa Rican homes, using the HBS methodology from the 2004 and 2013 national household surveys of income and expenditure [16], showed an increasing trend, and that it is therefore more conservative to assume it has not changed.

### 2.3 Counterfactual scenarios: Recommended salt intakes in the Costa Rican population

Recommended sodium intakes in Costa Rica are represented by two different sets of goals: The first is based on the "National Plan for the reduction of salt/sodium consumption in Costa Rica 2011–2021 [11]", and complies with the goal set by the WHO of reaching a salt intake of 5 g per person/day for an energy intake of 2000 kcal/d [7]. The second is based on the "National strategy for the comprehensive approach of non-communicable diseases and obesity 2014–2021" [13], which defines a 15% reduction in the average salt/sodium intake of the population.

Using both sets of recommendations, two counterfactual scenarios were established considering the current caloric intake of the overall population (Table 2):

- A very optimistic 46% reduction in salt intake (i.e. intake of 5.5±3.0g of salt per day for an energy intake of 2171 kcal), based on the recommendations of the “National Plan for the reduction of salt/sodium consumption in Costa Rica, 2011–2021”, which is the equivalent of an intake of 5 g per day for 2000 kcal as set by the WHO (scenario A).
- A more realistic 15% reduction in salt intake (i.e. intake of 8.5±4.5 g of salt per day for an energy intake of 2171 kcal), determined based on the “National strategy for the comprehensive approach of non-communicable diseases and obesity” (scenario B).

**Table 2:** Counterfactual scenarios: Recommended salt intakes in the Costa Rican population.

Salt intakes (g/day) at the average 2171 kcal intake of the Costa Rican population		
Baseline	Counterfactual scenarios (% reductions in salt intake)	
	Scenario A	Scenario B
	46%	15%
10.0±5.4	5.5±3.0	8.5±4.5

It was assumed that the Standard Deviations (SD) in the counterfactual scenarios represented the same proportion of the mean as in the baseline scenario.

### 2.4 Population demographics and mortality from CVDs

Age- and sex-specific data on population demographics and mortality were derived from the National Institute of Statistics and Census (INEC) of Costa Rica for 2018, since they are the most updated data and are intended to provide more recent information (S2 and S3 Tables).

The causes of death that are associated to salt consumption within PRIME, and that are therefore considered in the present study, included CVD according to the WHO-International Statistical Classification of Diseases and Related Health Problems [22]: Rheumatic heart disease (I05 to I09), Hypertensive disease (I10 to I15), Cerebrovascular disease (I60 to I69), Pulmonary embolism (I26), Heart failure (I50), Ischemic heart disease (I20 to I25), and Aortic Aneurysm (I71).

For the purpose of the analysis, the total number of deaths reported for 2018 was 23,026 and 5,649 of these were due to CVD.

### 2.5 Uncertainty analysis

The 95% Uncertainty Intervals (UI) of the estimates were calculated based on the 2.5 and 97.5th percentiles of results generated from 5000 interactions of a Monte Carlo analysis built-in PRIME [16]. The total deaths prevented or postponed (DPP) in the Costa Rican population were determined for the overall population as well as by sex and by cause (type of CVD) under each scenario tested.

## 3. Results

### 3.1 Total number of deaths prevented or postponed

In 2018, there were 5,649 deaths due to CVD in Costa Rica, which represented 24.5% of the total (n = 23,026). Fifty-five percent (55,5%; n = 3,136) of CVD deaths occurred among men and 44,5% (n = 2,514) occurred in women. Table 3 shows the estimates of the number of deaths due to CVD that would be prevented or postponed based on the two salt reduction scenarios.

**Table 3:** CVD deaths prevented or postponed (DPP) according to scenarios of salt intake reduction, Costa Rica 2018.

	DPP (95%UI )	
Sex	Scenario A^1^	Scenario B^2^
Men	418 (184–647)	164 (70–264)
Women	332 (147–513)	131 (55–210)
Total	750 (331–1160)	295 (125–473)

^1^ Scenario A: salt intake reduction by 46% (5.5±3.0 g/day) for a 2171 kcal/day intake, corresponding to an intake of 5 g/day for 2000 kcal as set by the WHO.

^2^ Scenario B: salt intake reduction by 15% (8.5±4.5 g/day) for a 2171 kcal/day intake.

According to the most modest scenario (B; 15% salt reduction), 5% (n = 295) of CVD deaths would be prevented or postponed, while in scenario A (46% salt reduction) they would reach 13% (n = 750) due to a higher reduction of salt intake. In absolute terms, the estimated number of DPP related to CVD is 2.5 times higher in scenario A than in scenario B. In both scenarios, only a slight difference was found between sexes.

## 3.2 Deaths prevented or postponed based on the type of CVD

The highest percentages of deaths that could be prevented or postponed according to the type of CVD in both scenarios would be related to Ischemic heart diseases (39% and 38% respectively for scenarios A and B), Hypertensive disease (32% and 33% respectively), and Cerebrovascular diseases (22% in both), as shown in Table 4. Salt reduction would only have a very low impact on deaths from Pulmonary embolism and Rheumatic heart disease (1% and 0% of DPP in both scenarios). Overall, the percentage of DPP for each type of CVD is similar between both scenarios. However, in absolute terms, the estimated number of DPP is 2.4 to 2.6 times higher in scenario A (46% reduction in salt consumption) than in scenario B (15% reduction) specifically for Ischemic heart diseases, Cerebrovascular diseases, Heart failure, Aortic aneurysm, and Hypertensive disease.

**Table 4:** CVD deaths prevented or postponed (DPP) according to scenarios of salt intake reduction, presented by type of cause^1^, Costa Rica 2018.

Cause	Scenario A^2^		Scenario B^3^	
	n of DPP (95%UI)	% of DPP	n of DPP (95%UI)	% of DPP
Ischemic heart diseases (I20 to I25)	290 (126–454)	39%	111 (47–178)	38%
Cerebrovascular disease (I60 to I69)	165 (72–259)	22%	65 (27–103)	22%
Heart failure (I50)	32 (14–52)	4%	12 (5–21)	4%
Aortic aneurysm (I71)	16 (7–26)	2%	6 (3–10)	2%
Pulmonary embolism (I26)	5 (2–10)	1%	2 (1–4)	1%
Rheumatic heart disease (I05 to I09)	3 (1–6)	0%	1 (0–2)	0%
Hypertensive disease (I10 to I15)	239 (106–367)	32%	98 (41–161)	33%
Total	750 (331–1160)	100%	295 (125–473)	100%

^1^ The codes within brackets correspond to the ICD codes.

^2^ Scenario A: salt intake reduction by 46% (5.5g/d/p±3.0) for a 2171kcal/day intake.

^3^ Scenario B: salt intake reduction by 15% (8.5g/d/p±4.5) for a 2171 kcal/day intake.

## 4. Discussion

This scenario modelling study showed that a considerable number of deaths attributable to CVDs could be prevented or postponed if the Costa Rican population reduced its dietary salt consumption by following the recommendations stated in the country. Despite the limited availability of recent data on nutrient intakes, including salt/sodium, at a population level, this is the first study about the health impact of salt intake reduction conducted in Costa Rica.

The results on the number of deaths attributable to CVDs that could be prevented or postponed are consistent with those found in countries such as Finland, the United Kingdom, Australia, and Ireland, where they have developed or are developing successful programs for the reduction of salt intake at the population level [1, 11, 20, 21, 23, 24]. Other countries that also used PRIME similarly showed that considerable proportions of deaths would be prevented or postponed with the reduction of salt intake (e.g. 23% in the UK and 10% in Canada) [25, 26].

Recent studies on sodium intake done in Mexico, Brazil, Nepal, and the USA [27–30] found important consumption differences between men and women. Men generally have a higher salt consumption, due to less healthy diets. This leads to the hypothesis that there could be differences between sexes in the number of deaths prevented or postponed following a salt reduction intervention. However, in the current study, there was a little difference in the number of DPPs between men and women mainly because HBS only provides the average salt intake of the overall population [21]. New studies that consider salt/sodium intake separately for both sexes would be required in Costa Rica to prove or discard this hypothesis.

High sodium intake (> 2 grams/day, equivalent to 5 grams of salt per day) and insufficient absorption of potassium (less than 3.5 grams per day) contribute to arterial hypertension and increase the risk of suffering from heart disease and cerebrovascular accident. The main benefit of reducing salt intake corresponds to the decrease of high blood pressure [31, 32]. In countries such as Finland, with strong policies towards salt reduction in the population since the ‘70s, a reduction of 3 g/day was accompanied by a reduction in high blood pressure and by a lessening of coronary diseases and cerebrovascular accident mortality by 75% - 80%, which increased life expectancy by 5 to 6 years [20, 24]. Interestingly, the current study demonstrated how salt intake reduction would save lives in relation to these three types of CVD (Ischemic heart disease, Hypertensive disease, and Cerebrovascular accident) in Costa Rican men and women. This aligns with a recent study carried out in the Nordic countries, where the impact of following the nutritional recommendations for preventing CVD mortality and cancer was modeled. This study concluded that Ischemic heart disease and CVD are related to most of the deaths that could be prevented or postponed by improving food intake towards healthier choices [18].

It should be noted that both men and women could obtain more benefits for their health after changing the intake of other components than sodium/salt in their diet, such as fruits, vegetables, legumes, fish, sugars, and fats [18]. Therefore, it would be interesting to use PRIME to determine the number of deaths related to CVD and other chronic diseases that could be prevented or postponed in association with changes in dietary factors different from sodium. However, as previously mentioned, there is no updated and official data in Costa Rica about the intake of these types of nutrients and food. The development of such a more complete type of analysis therefore remains pending the availability of data on other dietary factors.

The study has some limitations, including the shortage of official dietary intake data in Costa Rica. In the particular case of sodium, it must be taken into consideration that the HBS methodology exclusively provides information about food consumed at home. It therefore excludes all food eaten out of home, for example in restaurants, cafeterias, supermarket and other establishments [6, 24]. Nevertheless, the use of such data to determine consumption estimations in different populations is well accepted, with many studies having already used it for this purpose [6, 21]. Moreover, it estimates a significant percentage of total daily sodium consumption, since it considers processed foods, which are the second dietary source of sodium in the population of Costa Rica.

As indicated previously, the HBS does not allow for specific nutrient consumption estimations by age or sex, meaning that the same mean salt consumption was estimated for all age groups of the population. This could lead to biased salt intake estimations in the Costa Rican diet, as it has been suggested in other countries such as Brazil [31, 34].

Another limitation of the study is that the data used for salt consumption derives from the 2013 HBS (the most up-to-date data currently available) while the health benefits were estimated using year 2018 as a reference. Despite the fact that the latest studies on sodium intake from the previous ENN-2009 [11] and the latest analysis of the trend in sodium availability in Costa Rican households using the HBS methodology from the 2004 and 2013 national household surveys of income and expenditure [16], showed an increasing trend in sodium intake, a conservative approach was preferred, assuming that it has not changed since the last survey. The use of more recent and accurate data (24h urine) would provide an indication of whether sodium consumption remained stable or has changed.

Therefore, the estimates of the deaths prevented or postponed are likely an underestimation.

It is also important to mention that the recommendation established by the PAHO-WHO, regarding the maximum daily intake of sodium (2000 mg of sodium or 5 g of salt / day for 2000 kcal) and the established reduction goal in daily consumption (46%), are quite ideal and unlikely to be easily achieved in many countries like Costa Rica. Indeed, it must be taken into account that such reductions are related to various factors in the food environment and subject to multi-component interventions. This was one of the reasons why, in the “National Strategy for the Comprehensive Approach to Chronic Non-Communicable Diseases and Obesity 2014–2021”, a 15% relative reduction in the average salt/sodium intake of the population was targeted [13] and considered as a more realistic scenario to achieve in the medium and long term.

With regards to PRIME, all modelling equations related to changes in the diet and mortality originate from high-quality meta-analyses, which represents a strength. PRIME has also been used in several other studies in different countries, which denotes an additional strength [17]. However, PRIME only provides a cross-sectional analysis that indicates how many deaths would be avoided in a particular year. It does not provide estimation for a longer period, as with other models [17], while it is known that it might take several years to obtain the total health gains due to salt reduction [16–18, 31, 34]. Additionally, PRIME provides health benefits in terms of deaths prevented or postponed and does not quantify them as health gain in terms of Quality-Adjusted Life Years [QALY], which would have been useful to capture the quantity and the quality of gained lifespan, as well as the loss in productivity due to premature mortality, since the social consequences of the death of an individual over 65 years are different, for example, from that of a 45-year-old person [17].

Although PRIME considers the risk estimation by providing 95% uncertainty intervals through Monte Carlo simulations, allowing the associative parameters to vary stochastically according to distributions reported in the literature [17, 33, 34], the uncertainty proper to the HBS methodology or to the transformation of ingested food into nutrients still could not be accounted for in the model [17].

Lastly, PRIME models the DPP from a total reduction of salt in the diet, without considering its different food sources. In Costa Rica, the main source of dietary sodium is discretionary salt (60% of intakes) [6], as opposed to processed foods, which represent the main source in developed countries [1]. Therefore, it would be interesting in future studies to estimate the changes in DPP based on interventions specific to the characteristics of the country under study, such as interventions towards the reduction of the use of domestic salt.

## 5. Conclusions

This study evaluated the general impact on CVD mortality of modifying dietary salt intake at a population level in Costa Rica, and showed that reducing its consumption could prevent or postpone an important number of deaths. This study is the first of its kind in the country and is of utmost importance for public health authorities. Indeed, it provides reliable and useful estimations related to health and deaths that would be prevented or delayed following an intervention to reduce excessive salt consumption. Similarly, it evidences the urgent need to strengthen the implementation, follow-up, and evaluation of the “National Plan for the Reduction of Salt/Sodium Consumption in Costa Rica 2011–2021”, by the governing health bodies and involved institutions.

The findings support the importance of establishing a surveillance, monitoring, and evaluation program for salt/sodium consumption and other critical nutrients identified as dietary risk factors for NCDs. It also supports the importance of adopting other public policy measures that contribute to generate a healthy food environment in the country, including universal and mandatory nutritional labeling. Such measures would facilitate healthy decision-making by the population and reduce the epidemiological burden caused by an increased consumption of salt/sodium and other nutrients of public health interest.

## Supporting information

S1 Table Sodium available in Costa Rican households and caloric intake of the population from 2012 to 2013, using the Household Budget Survey analysis methodology (Grouped and ungrouped data by sodium (mg/day) and salt (g/day)). (DOCX)

S2 Table Costa Rican population over 15 years old, by sex and age groups, in 2018. (DOCX)

S3 Table Total CVD deaths in the Costa Rican population over 15 years old, by sex and age group, 2018. (DOCX)
