# Supplementary material for: Impact of salt intake reduction on CVD mortality in Costa Rica: A scenario modelling study
Source: PLoS One. 2021 Jan 12;16(1):e0245388. doi: 10.1371/journal.pone.0245388 (PMC7802917; doi:10.1371/journal.pone.0245388)
Supplement: S1 Table — (DOCX) [file pone.0245388.s001.docx]

S1 Table. Sodium available in Costa Rican households and caloric intake of the population from 2012 to 2013, using the Household Budget Survey analysis methodology (Grouped and ungrouped data by sodium (mg/day) and salt (g/day))^1^.

| **Intake** | **Ungrouped Data** | | **Grouped Data** | |
| --- | --- | --- | --- | --- |
|  | **sodium (mg)** | **salt (g)** | **sodium (mg)** | **salt (g)** |
| Average | 4537.87 | 11.34 | 4020.47 | 10.05 |
| SD | ±8772.13 | ±21.93 | ±2161.59 | ±5.40 |
| Kcal | 2352.02 | 2352.02 | 2170.67 | 2170.67 |

^1^In the HBS methodology, the grouping is used since it is considered that the reference period for the collection of information is not sufficient to characterize the pattern of food acquisition in each household and, therefore, the groups of households are adopted as the study unit corresponding to the strata of the sample instead of the individual households studied. These strata are made up of homogeneous household units from the point of view of territorial dominance and the socioeconomic condition of families, studied uniformly during the four quarters of the year. Therefore, the sample weight of each study unit (household stratum) corresponds to the sum of the sample weights of the households that make up the stratum. For more information, see the reference number 21.
