# Supplementary material for: Impact of salt intake reduction on CVD mortality in Costa Rica: A scenario modelling study
Source: PLoS One. 2021 Jan 12;16(1):e0245388. doi: 10.1371/journal.pone.0245388 (PMC7802917; doi:10.1371/journal.pone.0245388)
Supplement: S2 Table — (DOCX) [file pone.0245388.s002.docx]

S2 Table. Costa Rican population over 15 years old, by sex and age groups, in 2018 ^1^.

| **Ages** | **Total** | **Men** | **Women** |
| --- | --- | --- | --- |
| **Total** | **3 891 715** | **1 953 727** | **1 937 988** |
| 15-19 | 394 328 | 202 375 | 191 953 |
| 20-24 | 427 190 | 219 569 | 207 621 |
| 25-29 | 448 201 | 231 836 | 216 365 |
| 30-34 | 445 139 | 228 489 | 216 650 |
| 35-39 | 388 110 | 196 858 | 191 252 |
| 40-44 | 328 627 | 164 754 | 163 873 |
| 45-49 | 293 736 | 145 120 | 148 616 |
| 50-54 | 288 256 | 141 558 | 146 699 |
| 55-59 | 259 917 | 127 780 | 132 136 |
| 60-64 | 208 953 | 102 349 | 106 603 |
| 65-69 | 149 763 | 72 904 | 76 858 |
| 70-74 | 102 232 | 49 336 | 52 896 |
| 75-79 | 69 886 | 32 845 | 37 041 |
| 80-84 | 45 840 | 20 679 | 25 161 |
| 85 years old and over | 41 537 | 17 273 | 24 264 |

^1^Source: INEC, Population estimates and projections, 1950-2050
