# Supplementary material for: Impact of salt intake reduction on CVD mortality in Costa Rica: A scenario modelling study
Source: PLoS One. 2021 Jan 12;16(1):e0245388. doi: 10.1371/journal.pone.0245388 (PMC7802917; doi:10.1371/journal.pone.0245388)
Supplement: S3 Table — (DOCX) [file pone.0245388.s003.docx]

S3 Table. Total CVD deaths in the Costa Rican population over 15 years old, by sex and age group, 2018. ^1^

| Sex and age group (years) | |  | Death cause | | | | | | |
| --- | --- | --- | --- | --- | --- | --- | --- | --- | --- |
|  |  | **Total** | **Rheumatic heart disease** | **Hypertensive disease** | **Ischemic heart disease** | **Pulmonary embolism** | **Heart failure** | **Cardiovascular disease** | **Aortic aneurysm** |
|  |  |  | **I05 - I09** | **I10 - I15** | **I20 - I25** | **I26** | **I50** | **I60 - I69** | **I71** |
|  | **Total** | | | | | | | | |
|  | 15 - 19 | 3 | 0 | 0 | 0 | 2 | 0 | 1 | 0 |
|  | 20 - 24 | 4 | 0 | 0 | 2 | 0 | 0 | 2 | 0 |
|  | 25 - 29 | 20 | 1 | 1 | 9 | 1 | 2 | 6 | 0 |
|  | 30 - 34 | 25 | 1 | 2 | 12 | 2 | 0 | 6 | 2 |
|  | 35 - 39 | 44 | 1 | 4 | 23 | 1 | 1 | 13 | 1 |
|  | 40 - 44 | 80 | 2 | 6 | 48 | 5 | 1 | 14 | 4 |
|  | 45 - 49 | 105 | 1 | 9 | 53 | 5 | 3 | 28 | 6 |
|  | 50 - 54 | 197 | 1 | 13 | 119 | 7 | 7 | 45 | 5 |
|  | 55 - 59 | 281 | 5 | 20 | 179 | 2 | 9 | 58 | 8 |
|  | 60 - 64 | 405 | 6 | 42 | 261 | 0 | 11 | 79 | 6 |
|  | 65 - 69 | 440 | 7 | 42 | 271 | 8 | 12 | 83 | 17 |
|  | 70 - 74 | 532 | 4 | 77 | 274 | 6 | 23 | 131 | 17 |
|  | 75 - 79 | 700 | 2 | 86 | 367 | 13 | 28 | 186 | 18 |
|  | 80 - 84 | 834 | 6 | 135 | 397 | 8 | 41 | 229 | 18 |
|  | 85+ | 1979 | 11 | 384 | 857 | 17 | 121 | 555 | 34 |
|  | Total | 5649 | 48 | 821 | 2872 | 77 | 259 | 1436 | 136 |
|  | **Men** | | | | | | | | |
|  | 15 - 19 | 1 | 0 | 0 | 0 | 0 | 0 | 1 | 0 |
|  | 20 - 24 | 3 | 0 | 0 | 2 | 0 | 0 | 1 | 0 |
|  | 25 - 29 | 12 | 1 | 1 | 5 | 0 | 2 | 3 | 0 |
|  | 30 - 34 | 18 | 0 | 2 | 10 | 1 | 0 | 3 | 2 |
|  | 35 - 39 | 31 | 1 | 1 | 16 | 1 | 1 | 10 | 1 |
|  | 40 - 44 | 54 | 1 | 5 | 33 | 5 | 0 | 7 | 3 |
|  | 45 - 49 | 74 | 1 | 6 | 45 | 3 | 3 | 11 | 5 |
|  | 50 - 54 | 127 | 1 | 10 | 84 | 4 | 1 | 25 | 2 |
|  | 55 - 59 | 198 | 1 | 11 | 137 | 1 | 5 | 37 | 6 |
|  | 60 - 64 | 287 | 1 | 28 | 203 | 0 | 2 | 48 | 5 |
|  | 65 - 69 | 289 | 2 | 24 | 186 | 5 | 8 | 48 | 16 |
|  | 70 - 74 | 322 | 1 | 48 | 171 | 2 | 17 | 68 | 15 |
|  | 75 - 79 | 422 | 1 | 51 | 236 | 4 | 18 | 103 | 9 |
|  | 80 - 84 | 429 | 4 | 65 | 217 | 3 | 21 | 105 | 14 |
|  | 85+ | 869 | 4 | 160 | 412 | 6 | 51 | 214 | 22 |
|  | Total | 3136 | 19 | 412 | 1757 | 35 | 129 | 684 | 100 |
|  | **Women** | | | | | | | | |
|  | 15 - 19 | 2 | 0 | 0 | 0 | 2 | 0 | 0 | 0 |
|  | 20 - 24 | 1 | 0 | 0 | 0 | 0 | 0 | 1 | 0 |
|  | 25 - 29 | 8 | 0 | 0 | 4 | 1 | 0 | 3 | 0 |
|  | 30 –34 | 7 | 1 | 0 | 2 | 1 | 0 | 3 | 0 |
|  | 35 - 39 | 13 | 0 | 3 | 7 | 0 | 0 | 3 | 0 |
|  | 40 - 44 | 26 | 1 | 1 | 15 | 0 | 1 | 7 | 1 |
|  | 45 - 49 | 31 | 0 | 3 | 8 | 2 | 0 | 17 | 1 |
|  | 50 - 54 | 70 | 0 | 3 | 35 | 3 | 6 | 20 | 3 |
|  | 55 - 59 | 83 | 4 | 9 | 42 | 1 | 4 | 21 | 2 |
|  | 60 –64 | 118 | 5 | 14 | 58 | 0 | 9 | 31 | 1 |
|  | 65 - 69 | 151 | 5 | 18 | 85 | 3 | 4 | 35 | 1 |
|  | 70 - 74 | 210 | 3 | 29 | 103 | 4 | 6 | 63 | 2 |
|  | 75 - 79 | 278 | 1 | 35 | 131 | 9 | 10 | 83 | 9 |
|  | 80 - 84 | 405 | 2 | 70 | 180 | 5 | 20 | 124 | 4 |
|  | 85+ | 1110 | 7 | 224 | 445 | 11 | 70 | 341 | 12 |
|  | Total | 2513 | 29 | 409 | 1115 | 42 | 130 | 752 | 36 |

^1^ Source: Adapted of INEC, 2018 Deaths Statistics
